# Breast Cancer Mortality Hot Spots Among Black Women With de Novo Metastatic Breast Cancer

**DOI:** 10.1093/jncics/pkaa086

**Published:** 2020-10-01

**Authors:** Yunan Han, Marvin Langston, Lindsay Fuzzell, Saira Khan, Marquita W Lewis-Thames, Graham A Colditz, Justin Xavier Moore

**Affiliations:** 1 Division of Public Health Sciences, Department of Surgery, Washington University School of Medicine, St. Louis, MO, USA; 2 Department of Breast Surgery, First Hospital of China Medical University, Shenyang, China; 3 Division of Research, Kaiser Permanente, Northern California, Oakland, CA, USA; 4 Department of Health Outcomes & Behavior, H. Lee Moffitt Cancer Center, Tampa, FL, USA; 5 Epidemiology Program, College of Health Sciences, University of Delaware, Newark, DE, USA; 6 Department of Medical Social Sciences, Center for Community Health, Feinberg School of Medicine, Northwestern University, Chicago, IL, USA; 7 Alvin J. Siteman Cancer Center, Barnes-Jewish Hospital and Washington University School of Medicine, St. Louis, MO, USA; 8 Division of Epidemiology, Department of Population Health Sciences, Augusta University, Augusta, GA, USA

## Abstract

**Background:**

Black women living in southern states have the highest breast cancer mortality rate in the United States. The prognosis of de novo metastatic breast cancer is poor. Given these mortality rates, we are the first to link nationally representative data on breast cancer mortality hot spots (counties with high breast cancer mortality rates) with cancer mortality data in the United States and investigate the association of geographic breast cancer mortality hot spots with de novo metastatic breast cancer mortality among Black women.

**Methods:**

We identified 7292 Black women diagnosed with de novo metastatic breast cancer in Surveillance, Epidemiology, and End Results (SEER). The county-level characteristics were obtained from 2014 County Health Rankings and linked to SEER. We used Cox proportional hazards models to calculate adjusted hazard ratios (aHRs) and 95% confidence intervals (CIs) for mortality between hot spot and non–hot spot counties.

**Results:**

Among 7292 patients, 393 (5.4%) resided in breast cancer mortality hot spots. Women residing in hot spots had similar risks of breast cancer–specific mortality (aHR = 0.99, 95% CI = 0.85 to 1.15) and all-cause mortality (aHR = 0.97, 95% CI = 0.84 to 1.11) as women in non–hot spots after adjusting for individual and tumor-level factors and treatments. Additional adjustment for county-level characteristics did not impact mortality.

**Conclusion:**

Living in a breast cancer mortality hot spot was not associated with de novo metastatic breast cancer mortality among Black women. Future research should begin to examine variation in both individual and population-level determinants, as well as in molecular and genetic determinants that underlie the aggressive nature of de novo metastatic breast cancer.

Breast cancer is the most common cancer among women in the United States, with the highest incidence in White women. Yet, racial and ethnic disparities in breast cancer mortality rates persist (1,2). In the most recent period (2013-2017), the breast cancer mortality rate was 40% higher in Black women (28.4 per 100 000) vs White women (20.3 per 100 000) (2). Across all racial and ethnic groups, the stage of diagnosis is associated with survival. For women diagnosed with American Joint Committee on Cancer (AJCC)–defined stage I breast cancer, the expected 5-year breast cancer–specific survival rate is more than 96% (2). However, 5-year breast cancer–specific survival rates decrease as patients are diagnosed beyond AJCC stage I (3).

Approximately 5%-10% of all new breast cancer diagnoses are AJCC stage IV, also called de novo metastatic breast cancer (4,5). The overall prognosis for de novo metastatic breast cancer is poor, with the median overall survival ranging from 19 to 28 months (5,6); however, there have been recent improvements (4). These improvements may be partly attributed to advances in the molecular-level characterizations of breast cancer, use of hormone therapy, and targeted treatment strategies (7). Despite these improvements, breast cancer survival remains low among Black patients in late-stage distant metastatic breast cancer, according to The American Cancer Society’s "Breast Cancer Facts and Figures 2019-2020” (8).

As seen across racial and ethnic groups, geographic disparities in breast cancer outcomes exist across the United States (9). Historical factors, such as Jim Crow laws, housing and zoning policies, and migration patterns, have created swaths of racially segregated areas that still exist today (10,11). There has been an emergence of interdisciplinary researchers interested in the intersection of race and geographical location on health outcomes. Disease-specific geographic hot spots are the spatial aggregation of cases in an identifiable subpopulation based on geographic excess risks (12). A breast cancer mortality hot spot is a county identified as high risk for breast cancer mortality. Hot spots of breast cancer mortality among Black women were found primarily in rural southern counties near the Mississippi River and counties in the northern coastal North Carolina and southern Virginia areas of the United States (13).

However, to our knowledge, no study has investigated associations between geographic hot spots and mortality among Black women with de novo metastatic breast cancer. Thus, we are the first to link nationally representative data on breast cancer mortality hot spots with incident-based mortality data on de novo metastatic breast cancer in the United States and examine the association of geographic breast cancer mortality hot spots with de novo metastatic breast cancer mortality among Black women. Understanding this association can help medical and public health experts tailor programs and maximize resources to improve survivorship rates for this vulnerable group.

## Methods

### Study Design and Data

Data for this study were obtained from the Surveillance, Epidemiology, and End Results (SEER) 18 Registries Custom Data and the Centers for Disease Control and Prevention’s underlying causes of death file (14,15). This study was considered exempt by the institutional review board of Washington University School of Medicine because we used existing secondary data that are publicly available and nonidentifiable.

### Study Population

**Figure 1.**
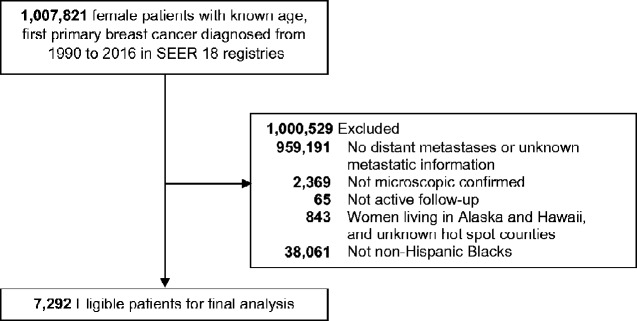
Flowchart of eligible individuals, SEER 18 registries 1990-2016. SEER = Surveillance, Epidemiology, and End Results.

We used “case listing” function through SEER*Stat software (version 8.3.6) to export potential cases for analysis from the SEER 18 database (16). Female patients with known age and diagnosed with first primary breast cancer from 1990 to 2016 were potentially eligible for this study (n = 1 007 821; Figure 1). Women were excluded if they had no distant metastases or unknown metastatic information (n = 959 191); lacked microscopic confirmation (n = 2369); were without active follow-up (n = 65); lived in Alaska, Hawaii, or unknown hot spot counties (n = 843); were not non-Hispanic Blacks (hereinafter, Blacks) (n = 38 061). Thus, our sample consisted of 7292 Black women with de novo metastatic breast cancer for final analysis. Individual demographic and clinical characteristics in SEER were used as potential covariates including age at diagnosis, year of diagnosis, marital status, tumor histology, tumor grade, hormone receptor status, the first course of treatments (surgery, radiation, and chemotherapy), and SEER registry. In brief, patients in metro counties with codes 1-3 were classified as urban, and all nonmetro counties with codes 4-9 were classified as rural.

### Hot Spots of Breast Cancer Mortality Among Black Women

Although a variety of approaches exist for spatial disease clustering, we used the aggregation of 3 separate local and global spatial clustering methods to identify counties that were hot spots for breast cancer mortality. This cluster identification method has been described in detail previously (13,17,18), and the use of this conservative approach helps avoid spurious results obtained from simply relying on one method. Briefly, we categorized county-level breast cancer mortality into 2 groups—hot spots or non–hot spots—based on a statistically significant higher observed vs expected breast cancer mortality rate. Hot spot counties are those identified as high risk for breast cancer mortality using all 3 approaches: 1) fifth quintile of smoothed Empirical Bayes breast cancer mortality rates, 2) high-high clusters using local indicators of spatial association, and 3) as a hot spot defined by Getis-Ord Gi* statistic (19,20). All other contiguous US counties were categorized as non–hot spots.

### County-Level Characteristics

To obtain characteristics on county-level demographic, socioeconomic, and health availability and/or community resources, cases identified in SEER were linked with county-level data from the 2014 County Health Rankings (CHR) and the 2014 American Community Survey (ACS) based on county Federal Information Processing Standards codes (21). The CHR and ACS comprise nationally representative data collected from a sample of the total noninstitutionalized population aged 18 years and older residing in households. The CHR database uses several survey samples to provide generalizable estimates of county-level factors, and the ACS provides aggregated estimates for demographic statistics over 5 years (2014 ACS, years 2010-2014). From CHR and ACS, we considered estimates of the county-level proportions of race and ethnicity, completed college, household income, obesity, smoking, excessive drinking, persons who could not see a doctor because of costs, limited access to healthy foods, mammography screening, physical inactivity, access to exercise opportunities, unemployment, uninsured, the ratio of primary care physicians per 100 000 persons, and rurality. County-level rurality was evaluated as a dichotomous variable (rural-urban) using 2010 Rural-Urban Commuting Area classifications, where urban areas were defined as population centers with more than 50 000+ residents, and rural and nonurban areas were defined as towns or small cities with population centers with fewer than 50 000 residents.

### Statistical Analysis

We compared bivariate differences in participant and tumor characteristics, county-level characteristics, and survival time in hot spots vs non–hot spot counties using χ^2^, analysis of variance, or Wilcoxon rank-sum tests as appropriate. We compared hot spots vs non–hot spot counties and presented the medians and interquartile ranges for the county-level characteristics because of the nonparametric distribution of continuous variables (Table 2). Because of the large number of counties examined, there were many associations considered statistically significant at α equals 0.05. Therefore, to observe the magnitude of the correlation between hot spots and county-level characteristics, we additionally examined the correlation of county-level characteristics with residents at diagnosis in county-level hot spots of breast cancer mortality using a Spearman correlation (*ρ*, positive values indicate positive correlation, and negative values indicate negative correlation). We examined the proportional hazards assumption for breast cancer–specific survival and overall survival by Schoenfeld residuals and by graphically assessing the log-log plots of survival. Therefore, we used Cox proportional hazards models to assess hazard ratios (HR) and 95% confidence intervals (CIs) for time to death from de novo metastatic breast cancer comparing hot spot and non–hot spot residence. Survival time was estimated from the date of diagnosis to the last date of follow-up or death. We performed a sequential model-building approach to examine possible confounders on the association between hot spot residence and mortality. The contribution of individual-level covariates, tumor factors, and treatments for multivariable models was assessed by a measure of the relative change in hazard ratios (22). Multivariable-adjusted model 1 was adjusted for individual-level factors (age, year of diagnosis, marital status, and SEER registries), tumor-level factors (tumor histology, tumor grade, and hormone receptor status), and treatments (surgery, chemotherapy, and radiation therapy). Multivariable-adjusted model 2 was adjusted for all factors in multivariable-adjusted model 1 with additional county-level factors that were associated with hot spots in a moderate Spearmen correlation (*ρ* ≥|0.20|). We performed statistical analyses using SAS version 9.4. All statistical tests were 2-sided, and *P* values less than  .05 were considered statistically significant.

**Table 2.** County-level characteristics by breast cancer mortality hot spot classification among 7292 Black women from SEER 18 registries, 1990-2016, linked with 2014 American Community Survey and County Health Rankings county-level data

County-level characteristics	Breast cancer mortality hot spot counties			
	Hot spot^a^ (n = 393; 5.4%)	Non–hot spot (n = 6899; 94.6%)	*P* ^b^	ρ^c^
	Median (IQR)^d^	Median (IQR)^d^		
% NH-White	59.3 (55.6-65.9)	46.6 (33.4-55.4)	<.001	0.17
% NH-Black	26.0 (18.5-36.6)	24.0 (8.4-40.0)	<.001	0.04
% Hispanic	5.0 (1.9-12.8)	9.1 (5.3-22.6)	<.001	−0.14
% Completed college	22.8 (13.3-25.8)	29.9 (21.7-39.4)	<.001	−0.21
% Household income <$20,000	20.3 (16.8-26.8)	18.5 (16.0-26.0)	<.001	0.08
% Obesity	33.5 (32.0-36.4)	26.9 (22.9-33.6)	<.001	0.23
% Smoking	20.8 (19.2-21.1)	14.8 (12.3-20.9)	<.001	0.20
% Excessive drinking	14.4 (12.0-17.2)	16.0 (15.0-17.7)	<.001	−0.05
% Could not see doctor because of cost	16.2 (13.6-17.6)	15.6 (13.5-18.1)	<.001	0.07
% Limited access to healthy foods^e^	5.0 (4.4-11.5)	4.0 (1.6-7.7)	<.001	0.16
% Mammography screening	61.4 (57.1-61.8)	59.3 (57.5-62.0)	.07	−0.02
% Physical inactivity	32.0 (27.8-32.9)	24.1 (19.6-27.7)	<.001	0.32
% Access to exercise opportunities	57.1 (40.2-88.8)	92.5 (81.2-96.8)	<.001	−0.25
% Unemployment	7.5 (6.2-10.6)	9.6 (8.6-10.9)	<.001	−0.19
% Uninsured	26.6 (19.4-27.9)	24.5 (21.5-28.5)	.59	0.01
PCP per 100 000 persons	6.7 (3.8-8.6)	7.1 (6.5-9.4)	.42	−0.12
% Nonurban (rural)	13.2 (1.6-24.2)	1.1 (0.6-5.9)	<.001	0.15

a Patients residing in counties with high breast cancer mortality (fulfilling all 3 criteria for geographic clustering). IQR = interquartile range; NH = non-Hispanic; PCP = primary care physicians; SEER = Surveillance, Epidemiology, and End Results.

b Statistical significance determined using χ^2^ tests for categorical variables or Wilcoxon rank-sum tests for nonparametric continuous variables.

c Spearman correlation with being a county-level breast cancer mortality hot spot.

d Median and IQR.

e Index that ranges from 0 (worst) to 10 (best), depending on the access to healthy foods by considering the distance an individual lives from a grocery store or supermarket.

## Results

### Descriptive Characteristics

Among 7292 Black women diagnosed with de novo metastatic breast cancer over the 27-year study period, approximately 5.4% (n = 393) women resided in hot spot counties at their diagnosis (Table 1). The mean age of all patients was 58.47 years and did not statistically significantly differ by hot spot classification (Table 1). Compared with women living in non–hot spot counties, women residing in hot spot counties were more likely to be diagnosed between 2010 and 2016 (52.9% vs 40.8%), more likely to reside in rural areas (18.3% vs 6.1%), more likely to have ductal histology of breast tumor (73.5% vs 67.7%), and more likely to have grade 2 tumor (30.5% vs 23.1%) (Table 1).

**Table 1.** Comparison of participant and tumor characteristics by breast cancer mortality hot spot classification among 7292 Black women diagnosed as de novo metastatic breast cancer from SEER 18 registries, 1990-2016

Characteristics	Total (n = 7292)	Breast cancer mortality hot spot counties		
		Hot spot^a^ (n = 393; 5.4%)	Non–hot spot (n = 6899; 94.6%)	*P* ^b^
Age at diagnosis, mean (SE), y	58.47 (0.17)	58.33 (0.72)	58.48 (0.17)	.95
Age at diagnosis, No. (%), y				
<40	706 (9.7)	39 (9.9)	667 (9.7)	.80
40-49	1246 (17.1)	65 (16.5)	1181 (17.1)	
50-59	1980 (27.2)	101 (25.7)	1879 (27.2)	
60-69	1699 (23.3)	102 (26.0)	1597 (23.2)	
70-79	1105 (15.2)	54 (13.7)	1051 (15.2)	
≥80	556 (7.6)	32 (8.1)	524 (7.6)	
Year of diagnosis, No. (%)				
1990-1999	1011 (13.9)	3 (0.8)	1008 (14.6)	<.001
2000-2009	3256 (44.7)	182 (46.3)	3074 (44.6)	
2010-2016	3025 (41.5)	208 (52.9)	2817 (40.8)	
Survival time, months, mean (SE)	23.72 (0.37)	21.59 (1.34)	23.84 (0.38)	.56
Marital status, No. (%)				
Single or never married	2582 (35.4)	134 (34.1)	2448 (35.5)	.38
Married or domestic partner	1940 (26.6)	100 (25.5)	1840 (26.7)	
Divorced, separated, or widowed	2383 (32.7)	131 (33.3)	2252 (32.6)	
Unknown	387 (5.3)	28 (7.1)	359 (5.2)	
Urban or rural residence, No. (%)				<.001
Urban	6801 (93.3)	321 (81.7)	6480 (93.9)	
Rural	491 (6.7)	72 (18.3)	419 (6.1)	
Tumor histology, No. (%)				
Ductal	4960 (68.0)	289 (73.5)	4671 (67.7)	.04
Lobular	541 (7.4)	31 (7.9)	510 (7.4)	
Mixed ductal and lobular	233 (3.2)	10 (2.5)	223 (3.2)	
Other histology	1558 (21.4)	63 (16.0)	1495 (21.7)	
Tumor grade, No. (%)				
1 (well differentiated)	251 (3.4)	17 (4.3)	234 (3.4)	.01
2 (moderately differentiated)	1716 (23.5)	120 (30.5)	1596 (23.1)	
3 (poorly differentiated)	3236 (44.4)	159 (40.5)	3077 (44.6)	
4 (undifferentiated)	135 (1.9)	7 (1.8)	128 (1.9)	
Unknown	1954 (26.8)	90 (22.9)	1864 (27.0)	
Hormone receptor status, No. (%)				
Positive (ER+ or PR+)	3913 (53.7)	205 (52.2)	3708 (53.8)	.61
Negative (ER- and PR-)	1993 (27.3)	116 (29.5)	1877 (27.2)	
Unknown	1386 (19.0)	72 (18.3)	1314 (19.1)	
Surgery, No. (%), yes	2541 (34.9)	136 (34.6)	2405 (34.9)	.18
Radiation, No. (%), yes	2339 (32.1)	111 (28.2)	2228 (32.3)	.09
Chemotherapy, No. (%), yes	4141 (56.8)	224 (57.0)	3917 (56.8)	.93

a Patients residing in counties with high breast cancer mortality (fulfilling all 3 criteria for geographic clustering). ER = estrogen receptor; PR = progesterone receptor; SEER = Surveillance, Epidemiology, and End Results.

b Statistical significance determined using χ^2^ tests for categorical variables, analysis of variance (ANOVA) for parametric continuous variables, or Wilcoxon rank-sum tests for nonparametric continuous variables.

County-level characteristics by hot spot classification are presented in Table 2. Black women living in hot spot counties with de novo metastatic breast cancer diagnosis were statistically significantly more likely than women living in non–hot spot counties to reside in areas with greater proportions of White residents (59.3% vs 46.6%), a greater prevalence of adult obesity (33.5% vs 26.9%), a greater prevalence of smoking (20.8% vs 14.8%), more physical inactivity (32.0% vs 24.1%), and a greater proportion of rural residents (13.2% vs 1.1%). Moreover, hot spot counties were statistically significantly less likely than non–hot spot counties to have greater proportions of Hispanic (5.0% vs 9.1%), to be areas with college completion rates (22.8% vs 29.9%), to have access to exercise opportunity (57.1% vs 92.5%), and less unemployment (7.5% vs 9.6%).

### Hot Spot Residence and Mortality

Over a median of 14 months, 5995 women (82.2%) died, including 5363 women (73.5%) from breast cancer (Table 3). Patients who resided in a breast cancer mortality hot spot were not at increased risk of death from de novo metastatic breast cancer mortality (adjusted hazard ratios [aHR] = 0.99, 95% CI = 0.86 to 1.13) compared with women living in non–hot spot counties, while controlling for individual-level factors, tumor-level factors, and treatments in multivariable model 1 (Table 3). We observed similar results after additionally adjusting for county-level proportions, including completed college education, population with obesity, current smoking status, physically inactive, and access to exercise opportunities, in multivariable model 2. Similarly, in fully adjusted models, women residing in hot spots had similar risks of breast cancer–specific mortality (aHR = 0.99, 95% CI = 0.85 to 1.15) and all-cause mortality (aHR = 0.97, 95% CI = 0.84 to 1.11) as women in non–hot spots after adjusting for individual and tumor-level factors and treatments (Table 3). Furthermore, the lack of association persisted when we evaluated different segments of follow-up time after disease diagnosis using piecewise Cox proportional hazards model controlling for full covariates in model 2 (0-2 years aHR = 1.05, 95% CI = 0.90 to 1.22; 2-4 years aHR = 1.20, 95% CI = 0.90 to 1.61; 4-6 years aHR = 1.23, 95% CI = 0.74 to 2.03; ≥6 years aHR = 0.95, 95% CI = 0.43 to 2.10; data not shown). Individual-level factors reduced the hazard ratio by 3.2%, treatments reduced the hazard ratio by 4.2%, and county-level factors reduced the hazard ratio by 1.6%, whereas tumor-level factors increased the hazard ratio by 2.7%. In total, adjustment for all covariates explained 6.2% of the breast cancer–specific survival across hot spot classification (Supplementary Table 2, available online). Additionally, the hazard ratios for overall mortality were similar to findings for breast cancer–specific mortality (Table 3).

**Table 3.** Multivariable hazard ratios for breast cancer–specific mortality and overall mortality among 7292 Black women diagnosis with de novo metastatic breast cancer from SEER 18 registries, 1990-2016

Breast cancer mortality hot spot	Breast cancer–specific mortality				Overall mortality			
	No. of breast cancer deaths/No. of women (%)^a^	Model 1 HR (95% CI)^b^	Model 2 HR (95% CI)^c^	Model 3 HR (95% CI)^d^	No. of all-cause deaths/No. of women (%)^a^	Model 1 HR (95% CI)^b^	Model 2 HR (95% CI)^c^	Model 3 HR (95% CI)^d^
Non–hot spot	5076/6899 (73.6)	Referent	Referent	Referent	5678/6899 (82.3)	Referent	Referent	Referent
Hot spot	287/393 (73.0)	0.99 (0.86 to 1.13)	0.97 (0.85 to 1.11)	0.99 (0.85 to 1.15)	317/393 (80.7)	0.98 (0.86 to 1.11)	0.96 (0.84 to 1.09)	0.97 (0.84 to 1.11)

a Numbers presented were from the Black women cohort (n = 7292). CI = confidence interval; HR = hazard ratio; SEER = Surveillance, Epidemiology, and End Results.

b Model 1 was adjusted for age at diagnosis, year of diagnosis, marital status, tumor histology, tumor grade, hormone receptor status, treatments (surgery, radiation, and chemotherapy), and SEER registry.

c Model 2 was additionally adjusted for county-level proportions for completed college education, population with obesity, current smoking status, physically inactive, and access to exercise opportunities in addition to model 1.

d Model 3 was adjusted for all individual-level, tumor-level, and county-level variables.

For comparison purposes, we examined the hazard ratios and 95% confidence intervals for all women in SEER 18 database from 1990 to 2016 (Supplementary Table 3, available online). Among 45 353 women, 30 967 were Whites (68.3%), 7292 were Blacks (16.1%), 4433 were Hispanic (9.8%), and 2661 were other races (5.9%). For this analysis, we identified 80 of 3108 (2.6%) counties as hot spots for breast cancer mortality for all women as our previous published article in mapping hot spots of breast cancer mortality in the United States (13). Among all women, we observed that residence in breast cancer mortality hot spots was not associated with increased risk of de novo metastatic breast cancer death (aHR = 1.06, 95% CI = 0.91 to 1.24; Supplementary Table 4, available online) after controlling for individual-level factors (age, race, marital status, and SEER registry). Compared with White women, Black women have a 30% increased risk of breast cancer–specific death (aHR = 1.30, 95% CI = 1.26 to 1.34; Supplementary Table 4, available online).

## Discussion

In this study, we are the first to link nationally representative data on breast cancer mortality hot spots with incident-based mortality data on de novo metastatic breast cancer among 7292 Black women in the United States. We observed that Black women diagnosed with de novo metastatic breast cancer had similar risk of death regardless of residence in county-level hot spots of breast cancer mortality.

The incidence of distant-stage breast cancer has increased over the past few decades (23). This increase may be partly explained by the more complete staging of advanced tumors (24) and increased use of advanced imaging to detect asymptomatic metastases (8). The prevalence of metastatic breast cancer in the United States from 1990 to 2020 was estimated that there will be 168 292 women living with metastatic breast cancer by 2020 (25). Evidence indicated that the odds of late-stage breast cancer among Black women were 43% higher when compared with their White counterparts (26) and demonstrated a widening racial and ethnic disparity gap in breast cancer stage at diagnosis in the recent decade (27-31). Further, studies using SEER data suggested that Black women may have up to a 45% increased risk of death from HER2-positive metastatic breast cancers when compared with White women (32). In comparison, another report of data from 2 breast-specific practices suggested that race and ethnicity may not contribute to the survival of patients diagnosed with de novo metastatic breast cancer (33). Differences in age distribution, race and ethnicity, comorbidities, and tumor factors could lead to survival differences between large SEER data and small practices. These mixed and limited studies support our exploration of breast cancer mortality hot spots and de novo metastatic breast cancer mortality among Black women, despite our null findings.

There is limited knowledge on both the geographic and racial patterns in de novo metastatic breast cancer in the United States. Several studies suggested an association between race and/or racial segregation with overall breast cancer outcomes. For instance, Bemanian and colleagues (34) observed that higher Black racial segregation was associated with a 9% lower risk of breast cancer–specific mortality among a sample of 940 Blacks diagnosed with invasive breast cancer. However, in a recent systematic review, Landrine and colleagues (35) found that living in segregated African American communities was associated with higher odds of late-stage breast cancer. Likewise, Pruitt and colleagues (36) reported that greater Black segregation was associated with a twofold increased risk of breast cancer–specific mortality; however, race and other factors explained away much of this association, with Black race being associated with a 46% increased risk of breast cancer mortality. Russell and colleagues (37) observed that among women in Georgia, segregation was associated with a twofold increased risk of death attributed to breast cancer for Black women, but not among White women. However, these racial segregation associations are complex because racial enclaves for Black women may lead to key survivorship and quality-of-life benefits such as increased social support, more culturally relevant care, more willingness to access care, and less exposure to stress from racism (34). To this end, because of the limited research surrounding the association between social determinants of health and de novo metastatic breast cancer specifically, future studies should continue to explore the social determinants of this disease and attempt to provide the geospatial distribution of metastatic breast cancer among the entire US population, which will provide insight into the highest areas of need for this increasing public health concern.

In our findings, we observed that among patients diagnosed with de novo metastatic breast cancer, there were no differences in breast cancer survival regardless of patients’ county of residence, even after adjustment for individual-level, tumor-level, and county-level variables. This could be due to other complex biology related to the aggressive phenotype of de novo metastatic breast cancer. Many cell cycle components contribute to a more aggressive tumor phenotype and poorer prognosis among Black breast cancer patients (38), such as higher mitotic index; overexpression of cyclin E, p16, p53; and lower expression of cyclin D1 (39). A better understanding of the key molecular determinants and genetic alterations that underlie clinical behavior in Black patients may also elucidate the higher mortality of de novo metastatic breast cancer.

Our study is strengthened by the use of nationally representative data to define mortality hot spots, as well as survival follow-up in SEER, which includes clinical and demographic data. Additionally, we were able to account for individual and population-level factors through multilevel modeling. However, the SEER database covers only 16 geographic areas within the contiguous United States of which only 5 areas (Iowa, New Mexico, Utah, Rural Georgia, and Kentucky) are considered rural. Last, SEER lacks data on comorbid conditions, where Black women were more likely than White women to have higher comorbidity scores (32).

Hot spots of breast cancer mortality for Black women were not associated with de novo metastatic breast cancer mortality. Our exploration adds to the limited evidence on geographic and racial patterns in metastatic breast cancer disease and mortality in Black women. Future research should begin to examine variation in both individual and population-level determinants, as well as in molecular and genetic determinants that underlie the aggressive nature of de novo metastatic breast cancer.

## Funding

Dr Colditz was supported by the Breast Cancer Research Foundation (award ID: BCRF-17-028) and the Alvin J. Siteman Cancer Center at Washington University School of Medicine and Barnes-Jewish Hospital in St. Louis, Missouri, the Biostatistics Shared Resource, Siteman Cancer Center, National Cancer Institute Cancer Center Support Grant no. P30 CA091842. Dr Langston was supported by an NIDDK UroEpi career development award K12DK111028. Dr Moore was supported by the National Institute on Minority Health and Health Disparities of the National Institutes of Health under award number K01MD015304

## Notes

**Role of the funders:** The funders had no role in the design of the study; the collection, analysis, and interpretation of the data; the writing of the manuscript; and the decision to submit the manuscript for publication.

**Disclosures:** The authors declare no potential conflicts of interest.

**Author contributions:** JXM conceived and designed the study. JXM and YH conducted the analysis. JXM and YH contributed to the interpretation of the data. YH, ML, LF, SK, and MWL drafted the manuscript, and all authors contributed to its critical review and editing.

## Data Availability

Patient data supporting all the tables and supplementary files in the published article will be made available on request from the corresponding author.

## Supplementary Material

pkaa086_Supplementary_Data
