# Supplementary material for: Breast Cancer Mortality Hot Spots Among Black Women With de Novo Metastatic Breast Cancer
Source: JNCI Cancer Spectr. 2020 Oct 1;5(1):pkaa086. doi: 10.1093/jncics/pkaa086 (PMC7791608; doi:10.1093/jncics/pkaa086)
Supplement: pkaa086_Supplementary_Data [file pkaa086_supplementary_data.pdf]

Supplementary Table 1. Percentage contribution of covariables to hazard ratios for breast cancer-specific mortality

Covariables	Cumulative % change of hazard ratio (HR)	% change of hazard ratio (HR)
Individual-level factors	3.19	3.19
Tumor-level factors	0.48	-2.71
Treatments	4.63	4.15
County-level factors	6.18	1.55
Total contribution, %	6.18	

NOTE. The contribution of individual-level factors, tumor factors, and treatments was assessed by a measure of the relative change. The relative change was defined simply as $[(D- - D+) \div D_0] \times 100$, in which D_0 is the hazard ratio (HR) from the age-adjusted Cox proportional hazards model, D- is the HR from the model without the covariables of interest, and D+ is the HR from the model with the covariables of interest. Covariables were added to the multivariable-adjusted models in the order stated. The percent change in overall differences attributable to a given covariable was adjusted for covariables added earlier in the sequence. A negative percent change indicates an increase in overall differences across hot spot classifications when the covariables are added to the model. The final cumulative percent change indicates the total proportion of overall survival differences across hot spot classifications explained by all covariables. The change in HR with the addition of covariables to the model is shown in Supplementary Table 3.

Supplementary Table 2. Hazard ratios for breast cancer-specific mortality with addition of covariables into multivariable models

Covariables	HR	95% CI
Baseline (age-adjusted)	1.04	0.92 to 1.17
Individual-level factors	1.00	0.88 to 1.14
Tumor-level factors	1.03	0.90 to 1.18
Treatments	0.99	0.86 to 1.13
County-level factors	0.97	0.85 to 1.11

Supplementary Table 3. Comparison of participant and clinical characteristics by breast cancer mortality hot spot classification among 45,353 women diagnosed as de novo metastatic breast cancer from SEER 18 registries, 1990-2016.

Characteristics	Total	Breast cancer mortality hot spot counties		
	N =45,353	Hot spot[a] N = 255 (0.56)	Non-hot spot N =45098 (99.44)	P-value[b]
Age at diagnosis, years, mean (SE)	61.82 (0.07)	64.05 (0.90)	61.81 (0.07)	0.01
Age at diagnosis, years, N (%)				
< 40	2932 (6.46)	11 (4.31)	2921 (6.48)	0.07
40-49	6403 (14.12)	33 (12.94)	6370 (14.12)	
50-59	10817 (23.85)	52 (20.39)	10765 (23.87)	
60-69	11109 (24.49)	59 (23.14)	11050 (24.50)	
70-79	8366 (18.45)	64 (25.10)	8302 (18.41)	
≥ 80	5726 (12.63)	36 (14.12)	5690 (12.62)	
Survival time, months, mean (SE)	29.75 (0.17)	23.80 (1.84)	29.78 (0.17)	0.01
Race and Ethnicity, N (%)				
NH-White	30967 (68.28)	181 (70.98)	30786 (68.26)	<.001
NH-Black	7292 (16.08)	59 (23.14)	7233 (16.04)	
Hispanic	4433 (9.77)	11 (4.31)	4422 (9.81)	
Other[c]	2661 (5.87)	4 (1.57)	2657 (5.89)	
Marital status, N (%)				
Single or never married	8977 (19.79)	26 (10.20)	2163 (19.85)	<.001
Married or domestic partner	19462 (42.91)	100 (39.22)	19362 (42.93)	
Divorced, separated, or widowed	14751 (32.52)	85 (33.33)	14666 (32.52)	
Unknown	2163 (4.77)	44 (17.25)	2119 (4.70)	
Tumor histology, N (%)				
Ductal	28827 (63.56)	168 (65.88)	28659 (63.55)	0.56
Lobular	4559 (10.05)	27 (10.59)	4532 (10.05)	
Mixed ductal and lobular	1999 (4.41)	7 (2.75)	1992 (4.42)	
Other histology	9968 (21.98)	53 (20.78)	9915 (21.99)	
Tumor grade, N (%)				
I (well differentiated)	2303 (5.08)	10 (3.92)	2293 (5.08)	0.31
II (moderately differentiated)	12653 (27.90)	73 (28.63)	12580 (27.89)	
III (poorly differentiated)	17299 (38.14)	92 (36.08)	17207 (38.15)	
IV (undifferentiated)	747 (1.65)	1 (0.39)	746 (1.65)	
Unknown	12351 (27.23)	79 (30.98)	12272 (27.21)	
Hormone receptor status, N (%)				
Positive (ER+ or PR+)	28139 (62.04)	151 (59.22)	27988 (62.06)	0.59
Negative (ER- and PR -)	9106 (20.08)	53 (20.78)	9053 (20.07)	
Unknown	8108 (17.88)	51 (20.00)	8057 (17.87)	
Surgery, yes, N (%)	16841 (37.13)	106 (41.57)	16735 (37.11)	0.33
Radiation, yes, N (%)	15182 (33.48)	82 (32.16)	15100 (33.48)	0.65
Chemotherapy, yes, N (%)	23859 (52.61)	128 (50.20)	23731 (52.62)	0.44

Abbreviations: ER, Estrogen receptor; HR, Hormone receptor; PR, progesterone receptor; SE, Standard error; SEER, Surveillance, Epidemiology, and End Results.

[a] Patients residing in counties with high breast cancer mortality (fulfilling all three criteria for geographic clustering).

[b] Significance determined using chi-square tests for categorical variables, analysis of variance (ANOVA) for parametric continuous variables, or Wilcoxon rank-sum tests for non-parametric continuous variables.

[c] Other race/ethnicity includes: American Indian/Alaska Native; Asian or Pacific Islander; or unknown.

Supplementary Table 4. Multivariable hazard ratios for breast cancer-specific mortality and all-causes mortality among 45,353 women diagnosis with de novo metastatic breast cancer from SEER 18 registries, 1990-2016.

	No. deaths (%) or mean (SE)[a]	Breast cancer-specific mortality	All-cause mortality
		HR (95% CI) [b]	HR (95% CI) [b]
Breast cancer mortality hot spot			
Non-significant	31475 (69.79)	Referent	Referent
Hot spot	172 (67.45)	1.06 (0.91- 1.24)	1.07 (0.93- 1.23)
Race and ethnicity			
NH-White	21841 (70.53)	Referent	Referent
NH-Black	5363 (73.55)	1.30 (1.26- 1.34)	1.32 (1.28- 1.36)
Hispanic	2876 (64.88)	1.00 (0.96- 1.04)	1.01 (0.98- 1.05)
Others	1567 (58.89)	0.89 (0.85- 0.94)	0.92 (0.87- 0.96)

Abbreviations: HR, Hazard Ratios; SEER, Surveillance, Epidemiology, and End Results.

[a] Presented as number of deaths and row (strata) proportion or mean and standard error for continuous variables.

[b] Adjusted for age (continuous), race and ethnicity, marital status, SEER registry.
